# Needle-Free Jet Injectors and Their Potential Applications in Plastic Surgery: A Review

**DOI:** 10.1093/asjof/ojaf019

**Published:** 2025-04-02

**Authors:** Neil M Vranis, Spero Theodorou

## Abstract

Needle-free jet injector (NFJI) devices offer an alternative to traditional needle–syringe injection for administration of medications, vaccines, and other fluids. They are already in clinical use around the world for vaccines and insulin administration. NFJIs have reproducibly demonstrated improvement in speed of drug absorption and efficacy; however, this technology has yet to prevail in the plastic surgery industry. Plastic surgeons have experimented with this technology to inject botulinum toxin for palmar hyperhidrosis, steroids for keloids/hypertrophic scars, and local anesthesia. A review was conducted to identify studies published in the last 10 years, indexed on PubMed, involving NFJI devices. Original articles that included ex vivo experimentation, animal studies, and human trials were included. Review articles, mathematical modeling theoretical articles, and nonmedical applications of jet injectors were excluded. A total of 767 identified articles were screened. Once exclusion criteria were applied, there were 115 articles remaining. The majority of the studies were conducted on human patients (*n* = 62, 53.9%), followed by ex vivo, small, and large animal models. Subcategories of investigations included device efficacy, mechanics, safety, and patient preferences. In conclusion, NFJI are a viable, safe, and potentially more efficacious delivery system compared with traditional needle–syringe techniques for the delivery of medications, vaccines, and other injectable fluids. Patient preference and pain were improved with NFJI. These devices have significant potential clinical applications in the plastic and dermatologic fields.

**Level of Evidence: 3 (Therapeutic):**

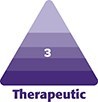

Medications, hormones, vaccines, anesthetics, and other medicinal injectables are deposited intradermally, subcutaneous or intramuscularly to optimize efficacy based on pharmacokinetics, pharmacodynamics, and indication for use. With traditional hypodermic needle–syringe techniques, efficacy and reproducibility are heavily influenced by injector skill and technique. Certain medications administered on a daily or weekly basis are often done at home through self-injection which can be particularly challenging for patients with needle phobia. Also, medication administration during high-stress/high-pressure scenarios (ie, intramuscular epinephrine injections for anaphylaxis) poses additional obstacles in precision of delivery. Needle–pen auto-injector devices were developed to account for some of these challenges by having predetermined needle lengths and dose delivery in order to minimize user error. However, a needle is still central to the device which is a significant drawback for patients with needle phobia.

The first jet injector was invented in 1860, in France, and works by forcing a stream of liquid through a precision nozzle to create a liquid jet that penetrates the skin and subcutaneous tissue.^1^ Needle-free jet injector (NFJI) devices were developed as an alternative to traditional needle injection methods of drug delivery. The lack of a needle is comforting to patients and avoids the risks inherent to sharp instruments. NFJI devices utilize an energy source to accelerate a piston that generates extremely high pressures to ultimately eject a high-velocity, narrow stream of fluid or powder from the nozzle. Medications/fluids are able to permeate the dermis and can be precisely delivered intradermally, subcutaneously, or intramuscularly by varying jet-stream velocities. Historically, these needle-free devices were developed for mass vaccination campaigns. However, the risk of spreading communicable diseases (including hepatitis) because of “splash back” and the inability to sterilize or dispose of the nozzle halted its acceptance.^2^ Several decades later, disposable nozzle/cartridges and single-use NFJIs were developed to minimize the potential of transmitting disease.^2^

Device mechanics ultimately determine fluid ejection velocity, which will the determine depth of penetration and medication dispersion. Once a threshold velocity is achieved, the fluid is able to pierce into or past the dermal layer. These devices were initially propelled by spring-loaded or gas-powered pneumatic systems. More recently, technology has advanced to include pyro-powered, laser-based, and Lorentz-force NFJI. These injectors can more precisely modulate piston pressure and exit velocity, which translates to improved control over depth of penetration and spread. The ability to accurately manipulate these variables enhances the utility and potential applications. The goal of this review was to evaluate the literature pertaining to NFJI, with a focus on their use and clinical applications.

## METHODS

A review was conducted on September 1, 2024, to identify all peer-reviewed, scientific articles involving NFJI devices for medical indications that were published in the last 10 years and indexed on PubMed. The following search parameters were applied: “((((jet) OR (pneumatic)) OR (needle-free)) OR (needle free)) AND (injector).” Exclusion criteria were based on abstract review and consisted of articles published outside of the last 10 years (before 2014), nonclinical injection purposes, nonmedical jet injectors, abstracts that mention NFJI but were not utilized in the study, review articles, and letters to the editor. Articles that only contain mathematical modeling were excluded. Ones that applied, mathematical modeling to laboratory experimentation was included.

Full articles were reviewed by both authors, and pertinent information was extracted. Data collected included title, first author, year of publication, journal of publication, type of NFJI utilized, and findings/results of the article. Any disagreements were discussed and resolved based on the senior author's (S.T.) judgment. Basic descriptive statistics were applied to report the findings.

## RESULTS

The search yielded 767 results. Two hundred and thirty-nine results (31.1%) were published within the last 10 years. After applying other exclusion criteria: isolated theoretical/mathematical modeling (*n* = 27), review articles (*n* = 43), letters to the editor (*n* = 4), mention of NFJI but not included in the study (*n* = 3), use of NFJI for other medical purposes (ie, capillary blood draws, cellular injection, cost–benefit analysis, air bolus delivery, and blood flow modeling; *n* = 13), and description of nonclinical jet injectors (ie, industrial engines, jet fuel systems, laboratory mass spectrometry, crystallographers, and electrophoresis; *n* = 33), there were 115 articles remaining for analysis.

Of the 115 articles that met inclusion criteria, there was a mean of 11.1 publications per year (range, 2-17). Seven studies did not specify which type of NFJI was used. The remaining studies involved spring mechanics (*n* = 40, 37.0%), pneumatic propulsion (*n* = 29, 26.9%), spring and pneumatic (*n* = 7, 6.5%), pyro-based (*n* = 13, 12.0%), Lorentz-force actuator (*n* = 9, 8.3%), laser based (*n* = 8, 7.4%), screw rotation (*n* = 1, 0.9%), and piezoelectric (*n* = 1, 0.9%). Three studies coupled an ex vivo study with an animal/human trial. The majority of studies involved human clinical trials (*n* = 62, 53.9%), followed by ex vivo studies (porcine or human skin samples; *n* = 25, 21.7%), small animal studies (rat, mouse, and chicken; *n* = 18, 15.7%), and large animal studies (monkeys, pigs, and goats; *n* = 13, 11.3%). The majority of the studies evaluated NFJI efficacy (*n* = 62, 53.9%), followed by safety and efficacy (*n* = 20, 17.4%), device mechanics (*n* = 16, 13.9%), safety (*n* = 12, 10.4%), mechanics and efficacy (*n* = 3, 2.6%), and patient preferences (*n* = 2, 1.7%; Supplemental Table).

## DISCUSSION

### Needle-Free Jet Injector Devices and Mechanics

**Figure 1.**
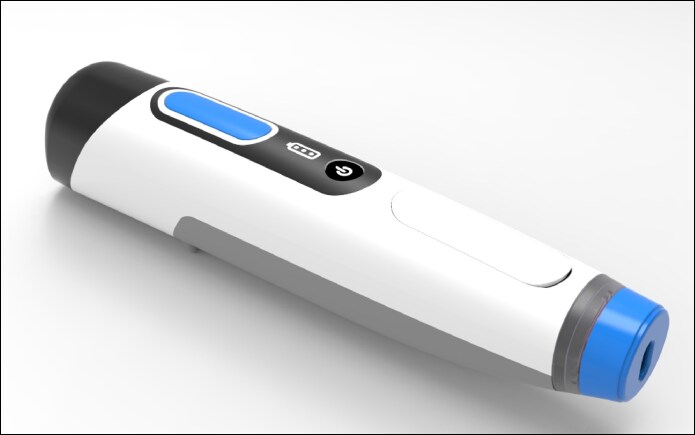
An example of a hand-held Lorentz-force actuator needle-free jet injector (PRIME, Portal Instruments, Cambridge, MA) Needle-free drug delivery system, which is approximately the size of a razor.

**Figure 2.**
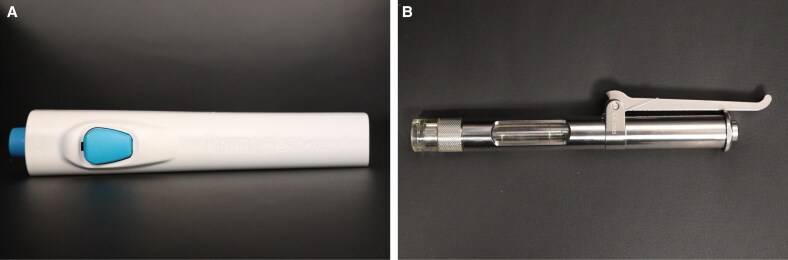
Examples of a hand-held, spring-loaded needle-free jet injectors. (A) Stratis (PharmaJet, Golden, CO). (B) MadaJet (Mada Medical Products, Inc., Carlstadt, NJ) stainless-steel injector.

NFJI devices (Figures 1, 2) are currently classified based on their power source. They are driven by spring mechanics, screw-rotary actuators, pneumatic gas (CO_2_, compressed air), pyro-driven electromagnetic Lorentz-force, piezoelectric motors, or laser based. Regardless of the power source, a conversion to kinetic energy is required in order to propel a substance from the cartage chamber through the nozzle and into or deep to the dermis without the use of a needle. Each of these power sources has their advantages and limitations, which are outlined in Table 1. The 3 main variables that determine depth of penetration and extent of distribution in tissue are: jet velocity, jet diameter, and substance viscocity.^3^ For the delivery of powder across the skin barrier, additional variables to consider are the density and radius of each particle.^4^ Typically, nozzle diameters range from 30 to 300 microns.

**Table 1.** The Benefits and Limitations of Various Types of NFJI

	Benefits	Deficiencies	Physics principle
Spring-loaded NFJI	Simple mechanics Low production cost Small handpiece	Nonadjustable velocity settings Nonadjustable volume settings Single dose delivered	Hooke's Law
Pneumatic NFJI	Adjustable velocity setting Adjustable volume settings Can deliver multiple doses	Larger hand piece Usually Requires hose to gas source Higher cost	Gas compression
Pyro NFJI	Adjustable velocity setting Adjustable volume settings	Single dose delivery for each combustion	Combustion energy of explosive
Lorentz-force actuator NFJI	Able to modulate pressures during injection (high pressure to penetrate tissue, lower pressure to deliver drug) Can deliver multiple doses	Typically, bulky and larger devices	Lorentz-force
Laser-based NFJI	Can generate extremely high velocities (up to 850 m/s) Rapid delivery of serial injections	Expensive Loud/noisy Heavy/bulky devices	Laser induced cavitation (wavelength and chromophore absorption)

**Figure 3.**
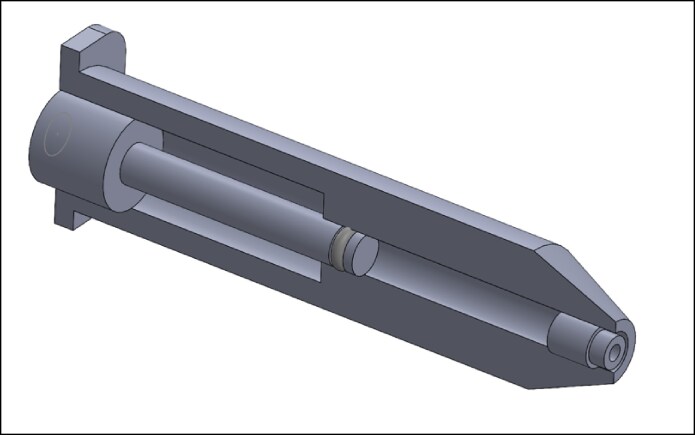
Illustration of a stainless-steel microjet cartridge consisting of a 4 mm bore and a 100 NFJI micron nozzle and a stainless-steel plunger with an *o*-ring. NFJI, needle-free jet injector.

Fluid dynamic mathematical modeling that accounts for fluid density, fluid compressibility, volume, piston acceleration, and cross-sectional area of the nozzle (Figure 3), accurately predicts the liquid ejection velocity of an NFJI. In general, jet velocity will increase as the plunger pressure increases, nozzle diameter decreases, and/or injectable material becomes less viscous.^15,16^ Exit pressures and velocity of the fluid correlate to the depth of penetration. But one of the most important variables is the collinearity of flow, which translates to improved penetration efficiency.^17^ Medical grade NFJI is able to generate high-speed fluid ejection achieving velocities from 100 to 350 m/s^18^ that can penetrate the dermis at variable depths and even reach the subcutaneous space or muscular regions. Studies confirm that 16 to 20 MPa of pressure is required to penetrate the stratum corneum, which translates to a jet velocity of at least 70 to 80 m/s in order to penetrate human skin.^5^

Simple, spring-loaded devices achieve a single shot velocity, whereas dual hydraulic and pneumatic NFJI devices were designed to accomplish the challenging endeavor of repeat injections at variable volumes and depths. Biphasic pyro- (combustion) and Lorentz-based (electromagnetic currents) NFJIs generate higher initial pressures for the penetration phase and a subsequent lower pressure for the dispersion phase in order to more accurately achieve a particular injection depth. An alternative method to minimize energy requirements without sacrificing the amount of fluid delivered is to manipulate the ampule shape. Creating ampules with 2 concentric pistons of different diameters will generate 2 phases of jet speeds despite a constant force from the piston. Harnessing more efficient power from the source ultimately leads to the possibility of making devices smaller and more lightweight, which is one of the drawbacks of the more complex systems. Velocity of fluid ejection was correlated with depth of penetration without change in distribution width.^5^

### Needle-Free Jet Injector Safety

The perception of pain has been found to be directly related to the diameter of skin trauma. Even with conventional needle injections, the smaller diameter needles (higher gauge) have decreased skin trauma, and thus, patients experience less pain. Depending on the nozzle diameter and velocity combination, fluid can be delivered through a skin hole that is 1/3 to 1/4 the diameter of traditional insulin pen needles. The skin entry hole of an NFJI can be 0.17 mm, which is significantly smaller than a 30 G needle (0.31 mm).^6^ Perceived pain also includes a component of anxiety associated with needles. In a study of 10 patients, the authors found that there was significantly less pain, utilizing a validated Patient and Observer Scar Assessment Scale (POSAS) score, associated with delivery of steroids to keloids when the NFJI was used compared with traditional needle injection techniques.^1^ These findings were confirmed in many studies that also utilized validated pain scales (POSAS, Numeric Pain Rating Scale, and Visual Analog Scale). All found a significant decrease in pain when comparing NFJI to traditional needle–syringe injection.^7,8^ Certain medical conditions (diabetes, relapsing-remitting multiple sclerosis, rheumatoid arthritis, allergies, growth hormone deficiency) require home administration self-injection of medications and hormones. NFJI devices may allow for improved medication compliance in such instances.^8^

Additionally, inadvertent needle-stick injuries are common in the healthcare profession. Particularly, in emergency situations (ie, seizures), needle-free devices provide an additional level of protection for providers and the patient. Repetitive, high-volume injection situations (ie, vaccination campaigns) are also high risk for providers. Because of its increased safety, 91% of injectors preferred NFJI over traditional techniques for mass polio vaccination.^9^

### Impact on Injectable Material

Given the high pressure generated from the chamber and the high speed of collision with the dermis, skeptics initially questioned the potential for unpredictable/inadequate dose delivery and deformation of substance. However, the sheer force and trauma caused by needle injection are more damaging to molecules.^10^ NFJIs have been used for animal vaccinations, human vaccinations, administration of insulin, anesthetic, and even botulinum toxin for palmar hyperhidrosis.^11^ Multiple studies have confirmed that even the most sensitive substances, such as DNA plasmids, mRNA, liposomes, and nanosuspensions, can be successfully propelled across the dermis with maintenance of their physical and functional properties.

One drawback of NFJI is the potential for the generation of small-droplet particle (<5 micron) aerosolization regardless of device mechanics. When ejection velocities are optimized for the substance and the dermal thickness, vaccines can be successfully administered in clinical studies with minimal splashback, aerosolization, or waste.^12^ In terms of dose efficiency, because of the inherent minimal “deadspace” since there is no needle, NFJI were deemed to be highly efficient in comparison, delivering the entire dose in a vial, with minimal waste. Table 2 outlines some of the benefits and limitations of NFJI.

**Table 2.** The Clinical Benefits and Limitations of Utilizing an NFJI

Benefits
Reduced risk of needle-sticks, improved operator safety Reduced risk of needle sharing with improved infection control Reduced anxiety for patients who have needle phobia Ability for mass pharmaceutical delivery (ie, vaccine administration) Rapid execution of procedure Higher accuracy of predetermined injection depths (compared with traditional hand-held needle injections) Can generate a more even distribution of medication Privacy and comfort of home injections Drug delivery consistency is less reliant on user technique, experience and skill with a needle and syringe Avoidance of needle blunting with serial injection sites
Drawbacks
Potential for dermal trauma (epidermal cysts, prolonged erythema) Potential for “splashback” (infection risk, loss of drug substance) Potential for generation of aerosolized small-droplet particles Inadequate dose delivery with incomplete dermal penetration Device malfunctions (clogging of nozzle, insufficient propulsive pressure generated) More complicated formula for determining injection depth and volume of delivery

NFJI, needle-free jet injector.

### Needle-Free Jet Injector Efficacy and Clinical Application

Thus far, substances that have been tested or approved for use by NFJI somewhere in the world include: animal vaccines, human vaccines, epinephrine, proteins, saline, chemotherapeutic agents, hormones, local anesthetics, exosomes, and other medications. Administration of insulin was one of the first substances to be delivered through an NFJI in human patients, which dates back to 1941.^13^ Compared with traditional needle–syringe injection or needle–pen injection, insulin administered through an NFJI has a wider subcutaneous distribution, which translates to a more rapid absorption into the bloodstream thereby reducing postprandial hyperglycemic fluctuations.^13^ Multiple studies comparing insulin and glucose blood levels after injection by NFJI vs traditional insulin pens confirmed that there was consistently better blood glucose control with the NFJI.^14,15^ Faster onset, accelerated insulin absorption and improved immediate glycemic control translate to slower disease progression and statistically significant decreases in hemoglobin A1c.

A similar pattern of clinical benefit has been observed with vaccines. Vaccines, such as ones for tuberculosis, that require intradermal deployment are more technically demanding. These are more consistently and reliably delivered by an NFJI compared with the traditional needle–syringe injection.^16^ The immunogenic efficiency of fractional (1/5th the dose) inactive polio vaccines delivered intradermal equaled full dose delivered intramuscularly. Thus, consistent delivery of intradermal dosing results in greater cost-effectiveness and therapeutic benefit. Immunogenicity, titers, and seropositive status improve with dermal injections compared with intramuscular injections given the higher density of native immune cells (antigen-presenting cells) present in the dermal layer.^17^ Vaccines without carrier proteins are also being evaluated for delivery by NFJI and proving to be clinically effective. Implications include a more cost-effective vaccine and injection of a pure vaccine without compromising on efficacy. Before NFJI, DNA-based vaccines would not elicit enough of a humoral/cellular response to induce clinical immunity. With the advent of NFJI, where injected material has a greater intradermal spread, the possibility of developing DNA-based vaccines that will induce adequate clinical immunity is promising.^18^

The ability to successfully reach an intramuscular target will also have several clinical implications for NFJI devices.^19^ A clinical trial comparing NFJI with traditional needle–syringe techniques for the intramuscular delivery of midazolam demonstrated similar bioequivalence, but a significantly faster onset when the medication was delivered through the NFJI.^19^ The authors advocate for the use of such devices when treating seizures, because time to resolution is neurologically critical for the patient. In circumstances of anaphylaxis, successful intramuscular injection is also imperative. A common site of injection is the anterolateral thigh. Using a pneumatic (gas) injector, the drug successfully reached the intramuscular region 94.6% of the time.^20^ The mean depth of penetration in this study was 3 cm; gender, skin thickness, and BMI did not influence the efficacy.^20^

### Role of Needle-Free Jet Injectors in Plastic Surgery

Plastic surgeons often treat keloids and hypertrophic scars. These pathologic scars showed objectively better improvements when steroids or bleomycin were delivered through NFJI compared with needle injections, and were deemed to be less painful.^21^ Similar to vaccines, a more precise biodistribution of steroid delivery into the keloids was demonstrated with NFJI compared with traditional needle–syringe methods which may account for the observed scar improvement. Because this technology continues to become more widespread, treatments of scars with NFJI will likely surpass the more traditional delivery methods. Treatment of palmar hyperhidrosis is also a part of a hand surgeon's or plastic surgeon's practice. Delivery of neurotoxin into the dermis minimizes risk of unintended diffusion to hand muscles that may compromise function. Neurotoxin was successfully injected into the hands of 20 patients without the use of anesthesia before NFJI, and outcomes were not inferior to traditional needle injection techniques.^22^ Time of treatment with NFJI was 5 min compared with 20 to 25 min.^22^ NFJI will greatly improve efficiency for procedures that require multiple injections.

Additionally, plastic surgeons are often called to perform emergency room procedures in children who require complete and successful anesthesia or sedation. In young children requiring sedation for bedside procedures, subcutaneous midazolam delivered by NFJI achieved the same level of sedation with higher parent satisfaction compared with intravenous midazolam.^23^

Although this technology has been around for decades, its use remained limited until recently. In the realm of facial rejuvenation, many centers around the world are employing NFJI to deliver collagen stimulating substances at specific dermal depths. Improvements in skin “darkness,” redness, texture, and dermal collagen/elastin were demonstrated (clinical rating and histology, respectively) in 27 female patients with high patient satisfaction after full face injections of poly-Dl-lactic acid (PDLA) mixed with hyaluronic acid through an NFJI.^24^ Exosomes, a novel treatment permitted in certain countries, injected by a pneumatic NFJI penetrated the dermis with histologic confirmation of reaching the subdermal tissue. Another group demonstrated significant improvement of striae distensae (“stretch marks”) in 4 patients after serial injection of PDLA with an NFJI.^25^ The ability to consistently deliver very small aliquots of a medication/product at precise depths in a tolerable and efficient manner has the potential to change injectable facial rejuvenation.

### Limitations

Certainly, limitations exist for this review. This study represents a broad overview of the literature that has been published in the last 10 years regarding NFJI. Although it is not all-encompassing, it does represent approximately one-third of studies that have been published on the topic. Additionally, we chose this time period because it reflects the most current and relevant research. Given the broad nature of this review, we are unable to cluster the data and perform a meta-analysis that would be objective and useful. Rather, this overview demonstrates the broad utilizations of this technology and future implications, particularly in the realm of plastic surgery.

## CONCLUSIONS

NFJI offers an alternative method for practitioners and patients to administer medications in a safe, reliable, and efficient method. The majority of studies demonstrated increased patient satisfaction, increased injector safety and decreased recipient pain with NFJI compared with the traditional delivery systems. The data suggest increased medication efficacy when the injectable medications are delivered through an NFJI. There is strong clinical data involving vaccine and insulin administration that substantiate these claims. The improved immunogenicity and glycemic control, respectively, will have significant clinical implications because these devices become more widely utilized. Additionally, NFJI offers the opportunity for patients with prohibitive needle phobia to be eligible for injectable therapeutics. Although spring and gas-based NFJI may be simple and cost-effective, the recent advances in Lorentz-force technology may allow the injector to more precisely control the depth of penetration and the volume of fluid delivered. The clinical applications in the medical and cosmetic realm will begin with topical anesthetics and perhaps botulinum toxin injections but are truly limitless.

## Supplementary Material

ojaf019_Supplementary_Data
