# Supplementary material for: Needle-Free Jet Injectors and Their Potential Applications in Plastic Surgery: A Review
Source: Aesthet Surg J Open Forum. 2025 Apr 2;7:ojaf019. doi: 10.1093/asjof/ojaf019 (PMC12065001; doi:10.1093/asjof/ojaf019)
Supplement: ojaf019_Supplementary_Data [file ojaf019_supplementary_data.docx]

| ***Title*** | ***Authors*** | ***Year*** | ***Journal*** | ***Device Mechanics*** | ***Research Model*** | ***Sub-Topic*** | ***Outcome*** |
| --- | --- | --- | --- | --- | --- | --- | --- |
| Needle-Free Injection of Exosomes Derived from Human Dermal Fibroblast Spheroids Ameliorates Skin Photoaging | Hu et al. | 2019 | ACS Nano | Pneumatic | Small Animal (Mouse Model) | NFJI Efficacy | exosomes have skin rejuvenating properties |
| Needle-Free Jet Injectors and Nanosuspensions: Exploring the Potential of an Unexpected Pair | Schlich et al. | 2022 | Pharmaceutics | Spring (Comfort-In) | Porcine Skin, Small Animal (Rat Model) | NFJI Efficacy | nano-suspension injected without loss of product amount or function |
| A Needle-Free Jet Injection System for Controlled Release and Repeated Biopharmaceutical Delivery | Trimzi et al. | 2021 | Pharmaceutics | Pneumatic + Spring | Porcine Skin | NFJI Mechanics and Efficacy | theoretical variable volumes match actual volumes of injection, validating device |
| Rotatable Orifice for Needle-Free Jet Injection | Tan et al. | 2023 | Annual Int Conf IEEE Eng Med Biol Sci | Lorenz Force Actuator | Porcine Skin | NFJI Mechanics | o-ring creates the most efficient seal, amount of fluid delivered depends on angle |
| Poly‐d,l‐lactic acid‐enhanced atrophic scar treatment via transdermal microjet drug delivery in Asians | Seo et al. | 2024 | Skin Res Technol | Laser assisted (MiraJet) | Human (n=5) | NFJI Safety | mild discomfort reported |
| The Effectiveness of Jet (Needle-Free) Injector to Provide Anesthesia in Child Circumcision under Local Anesthesia | Akyuz et al. | 2022 | J Invest Surg | Spring (MadaJet XL) | Human (n=64) | NFJI Safety | Jet injector ring block did not achieve sufficient anesthetic level for pediatric circumcision |
| The Absorption of Needle-Free Insulin Aspart Through Jet Injector in Different Body Parts of Healthy Individuals | Pan et al. | 2022 | Front Endorcinol | Spring (Beijing QS) | Human (n=8) | NFJI Efficacy | Jet Injector at various anatomic areas (skin thickness different) did not change glucose curve |
| Needle-free electronically controlled jet injection with corticosteroids in recalcitrant keloid scars: a retrospective study and patient survey | Bik et al. | 2023 | Lasers Med Sci | Pneumatic | Human (n=10) | NFJI Safety | NFJI had lower pain scores compared to traditional needle injections |
| Therapeutic Effectiveness of Needle Injection Versus Needle-Free Jet Injector System for Botulinum Toxin Type A in Palmar Hyperhidrosis | Watanabe et al. | 2023 | J Cutan Med Surg | Pneumatic (Med-Jet) | Human (n=16) | NFJI Safety | Clinical no difference btw inj methods, less pain (visual analog score) |
| Needle-free jet injector intradermal delivery of fractional dose inactivated poliovirus vaccine: Association between injection quality and immunogenicity | Resik et al. | 2015 | Vaccine | Spring (Tropis) & Pneumatc (BioJect) | Human (n=729) | NFJI Efficacy | PharmaJet was more efficacious for vaccine (polio) delivery |
| Jet-injection assisted photodynamic therapy for superficial and nodular basal cell carcinoma: A pilot study | Lavin et al. | 2024 | Lasers Surg Med | Pneumatic (AirGent) | Human (n=15) | NFJI Efficacy | NFJI is viable alternative to deliver ALA for BCC (photosensitization therapy) with good clinical response |
| Spring-Powered Needle-Free Injection of Triamcinolone Acetonide and 5-Fluorouracil for Keloid Treatment | Sutedja et al. | 2023 | Clin Cosmet Investig Dermatol | Spring | Human (n=1) | NFJI Safety | NFJI was tolerable for 5-FU/Triamcinalone injection intralesional keloid using validated NPRS and POSAS (pain scales) |
| A clinical observation study on the effect of needle-free insulin syringe on blood glucose control and well-being index in patients with early-onset type 2 diabetes mellitus | Jin et al. | 2023 | Front Endocrinol | Spring (Beijing QS) | Human (n=42) | NFJI Efficacy | NFJI controlled blood glucose better than insulin pen. Lower pain score with NFJI, # red spots higher with NFJI |
| Needle-free injection of basal insulin improves fasting glucose variability as assessed by continuous glucose monitoring in T2DM: a prospective randomized multicenter open-label crossover study | Sun et al. | 2022 | Expert Opin Drug Deliv | Spring (Beijing QS) | Human (n=48) | NFJI Efficacy | NFJI controlled fasting glucose level better than traditional insulin pen |
| Comparison of Three Anaesthetic Options to Reduce Acute Pain Response in Kid Goats | Cuttance et al. | 2024 | J Appl Anim Welf Sci | Lorenz Force Actuator | Large Animal (Goat) | NFJI Efficacy | NFJI and NFJI-assisted delivery of Lidocaine improved analgesia compared to anesthetic topical cream and vapocoolant spray |
| A Comparison in Patient Comfort Using Conventional Syringe and Needleless Jet Anesthesia Technique in Periodontal Surgery-A Split-Mouth Randomized Clinical Trial | Shankar et al. | 2022 | Medicina | Spring | Human (n=30) | NFJI Safety | NFJI of local intraoral had less pain (VAS scale, VRS scale) and less anxiety (Beck's anxiety inventory) |
| Clinical endpoints of needle-free jet injector treatment: An in depth understanding of immediate skin responses | Bik et al. | 2022 | Lasers Surg Med | Pneumatic & Spring | Human Skin ex vivo | NFJI Safety | Methylene blue dye study of intradermal spread btw spring vs pneumatic |
| Intradermal administration of fractional doses of the inactivated poliovirus vaccine in a campaign: a pragmatic, open-label, non-inferiority trial in The Gambia | Bashorun et al. | 2022 | Lancet Glob Health | Spring (Tropis) | Human (n=2,720) | NFJI Efficacy | non-inferiority trial - NFJI delivered an adequate dose to achieve immunity for polio |
| Advances in subcutaneous injections: PRECISE II: a study of safety and subject preference for an innovative needle-free injection system | Kelley et al. | 2021 | Drug Deliv | Pneumatic (Prime) | Human (n=42) | NFJI Safety | Less pain (VAS Score) compared to needle injection |
| Needle-free jet injection of insulin glargine improves glycemic control in patients with type 2 diabetes mellitus: a study based on the flash glucose monitoring system | Kong et al. | 2021 | Expert Opin Drug Deliv | Spring (Beijing QS) | Human (n=66) | NFJI Efficacy | RCT of injection modalities. NFJI had a better glucose control and lower mean glucose levels |
| Comparison of glucose variability in patients with type 2 diabetes administrated glargine with needle-free jet injector and conventional insulin pen | Sun et al. | 2020 | Expert Opin Drug Deliv | Pneumatic | Human (n=26) | NFJI Efficacy | Observational study. NFJI had a lower mean glucose level compared to needle pen |
| Delivery Strategies for Skin: Comparison of Nanoliter Jets, Needles and Topical Solutions | Cu et al. | 2020 | Ann Biomed Eng | Laser assisted | Porcine Skin | NFJI Efficacy | Comparison Study (Topical, needle, NF) |
| Safety and immunogenicity of investigational seasonal influenza hemagglutinin DNA vaccine followed by trivalent inactivated vaccine administered intradermally or intramuscularly in healthy adults: An open-label randomized phase 1 clinical trial | Carter et al. | 2019 | Plos One | Pneumatic (Biojector) | Human (n=316) | NFJI Efficacy and Safety | DNA flu vaccine injected with NFJI. Found to have higher reactogenicity (pain, swelling, redness) |
| Efficacy and safety of a needle-free injector in Chinese patients with type 2 diabetes mellitus treated with basal insulin: a multicentre, prospective, randomised, crossover study | Xing et al. | 2019 | Expert Opin Drug Deliv | Spring (Beijing QS) | Human (n=62) | NFJI Efficacy and Safety | RCT - NFJI demonstrated better glucose control and less pain/anxiety of drug administration |
| Study Protocol for a Prospective, Multicenter, Randomized, Open-Label, Parallel-Group Clinical Trial Comparing the Efficacy and Safety of a Needle-Free Insulin Injector and a Conventional Insulin Pen in Controlling Blood Glucose Concentrations in Chinese Patients with Type 2 Diabetes Mellitus (The FREE Study) | Ji et al. | 2019 | Adv Ther | Spring (Beijing QS) | Human (n=420) | NFJI Efficacy and Safety | Pros Multi Cent Trial - Study protocol published |
| Needle-free delivery of influenza vaccine using the Med-Jet® H4 is efficient and elicits the same humoral and cellular responses as standard IM injection: A randomized trial | Shapiro et al. | 2019 | Vaccine | Pneumatic (Med-Jet) | Human (n=80) | NFJI Efficacy and Safety | Equally as efficacious (cellular and humoral immunity), slightly more pain/erythema local reactions compared to traditional needle based flu vaccine |
| DNA vaccine priming for seasonal influenza vaccine in children and adolescents 6 to 17 years of age: A phase 1 randomized clinical trial | Houser et al. | 2018 | PLos One | Pneumatic (Biojector) | Human (n=75) | NFJI Efficacy and Safety | Pediatric vaccine study DNA flu and trivalant inactive influenza vaccine were equally efficacious. More pain associated with NFJI of DNA vs needle/syringe of IIIV3 |
| Immunogenicity and safety of measles-mumps-rubella vaccine delivered by disposable-syringe jet injector in India: A randomized, parallel group, non-inferiority trial | Bavdekar et al. | 2018 | Vaccine | Spring (Stratis) | Human (n=340) | NFJI Efficacy and Safety | RCT of MMR vaccine for toddlers using NFJI vs needle. Same immunogenicity achieved. Slightly higher number of mild local adverse skin effects |
| Comparative effects of in ovo versus subcutaneous administration of the Marek's disease vaccine and pre-placement holding time on the processing yield of Ross 708 broilers | Peebles et al. | 2017 | Poult Sci | Pneumatic | Small Animal (Chicken Model) | NFJI Efficacy | Animal vaccination study - NFJI conferred a slight advantage in vaccination efficacy |
| Pharmacokinetic and Pharmacodynamic Variability of Insulin When Administered by Jet Injection | Engwerda et al. | 2017 | J Diabetes Sci Technol | Spring (Insujet) | Human (n=30) | NFJI Efficacy | NFJI with increased pharmacokinetics of rapid acting insulin and no change to pharmacodynamics |
| Safety and immunogenicity of a modified vaccinia Ankara vaccine using three immunization schedules and two modes of delivery: A randomized clinical non-inferiority trial | Jackson et al. | 2017 | Vaccine | Spring (Stratis) | Human (n=435) | NFJI Efficacy | Anarka vaccine. Non-inferiority was established for NFJI vs needle on the non-compressed vaccine schedule. Redness and edema more common with NFJI |
| Comparison of jet injector and insulin pen in controlling plasma glucose and insulin concentrations in type 2 diabetic patients | Guo et al. | 2017 | Medicine | Not Specified | Human (n=60) | NFJI Efficacy | Superior efficacy in insulin administration with higher blood insulin levels and lower blood glucose levels |
| Efficacy of a Novel Prefilled, Single-Use, Needle-Free Device (Zeneo®) in Achieving Intramuscular Agent Delivery: An Observational Study | Bardou et al. | 2017 | Adv Ther | Pneumatic (Zeneo) | Human (n=37) | NFJI Efficacy | Able to reproducibly achieve intramuscular injections, even up to 3cm deep. |
| Safety and immunogenicity of inactivated poliovirus vaccine when given with measles-rubella combined vaccine and yellow fever vaccine and when given via different administration routes: a phase 4, randomised, non-inferiority trial in The Gambia | Clarke et al. | 2016 | Lancet Glob Health | Spring (PharmaJet Stratis) | Human (1,504) | NFJI Efficacy | RCT - comparing types of vaccines administered. No safety concerns found with NFJI |
| Lispro administered by the QS-M Needle-Free Jet Injector generates an earlier insulin exposure | Hu et al. | 2016 | Expert Opin Drug Deliv | Spring (Beijing QS) | Human (n=18) | NFJI Efficacy | Rand Blinded Control Trial - Insulin administered with NFJI resulted in higher and faster levels of blood insulin and the inverse for glucose levels |
| Comparison of local anesthetic effect of lidocaine by jet injection vs needle infiltration in lumbar puncture | Hajimaghsoudi et al. | 2016 | Am J Emerg Med | Spring (Injex) | Human (n = 44) | NFJI Efficacy | NFJI of insulin for lumbar puncture was less painful than traditional needle infiltration using VAS pain score |
| Successful treatment of thyroidectomy scar with a pneumatic needleless injector and silicone gel | Seok et al. | 2016 | Int Wound J | Pneumatic (Innojector) | Human (n=1) | NFJI Efficacy | Case report of injecting saline with NFJI for scar improvement. |
| The effect of jet shape on jet injection | Park et al. | 2015 | Annual Int Conf IEEE Eng Med Biol Sci | Lorenz Force Actuator | Porcine Skin | NFJI Mechanics | Physics and mechanics of jet stream - collimated jet stream has greater penetration than dispersed jet stream |
| Depressed scar after filler injection successfully treated with pneumatic needleless injector and radiofrequency device | Seok et al. | 2015 | Dermatol Therap | Pneumatic (Innojector) | Human (n=1) | NFJI Efficacy | Improved depress scar appearance with RF device + NFJI |
| Insulin administered by needle-free jet injection corrects marked hyperglycaemia faster in overweight or obese patients with diabetes | Wit et al. | 2015 | Diabetes Obes Mtab | Spring (Insujet) | Human (n=26) | NFJI Efficacy | Random Controlled Crossover - NFJI improves speed of hyperglycemia correction compared to needle injectors |
| A randomized clinical trial in adults and newborns in South Africa to compare the safety and immunogenicity of bacille Calmette-Guérin (BCG) vaccine administration via a disposable-syringe jet injector to conventional technique with needle and syringe | Geldenhuys et al. | 2015 | Vaccine | Pneumatic (Biojector) | Human (n=66) | NFJI Efficacy and Safety | NFJI deposited a greater amount of intradermal BCG vaccine compared to traditional. No difference in adverse effects. |
| Immunogenicity and safety of measles-mumps-rubella vaccine delivered by disposable-syringe jet injector in healthy Brazilian infants: a randomized non-inferiority study | Martins et al. | 2015 | Contemp Clin Trials | Spring (PharmaJet) | Human (n=582) | NFJI Efficacy and Safety | Crying was more likely with needle stick, local adverse skin effects (mild) more common with NFJI. MM immunogenicity was inferior, but rubella was non-inferior |
| Immune responses after fractional doses of inactivated poliovirus vaccine using newly developed intradermal jet injectors: a randomized controlled trial in Cuba | Resik et al. | 2015 | Vaccine | Pneumatic (Biojector) & Spring (Intradermal Pen)/(Tropis) | Human (n=728) | NFJI Efficacy | One of two NFJI were able to consistently deliver intradermal polio vaccine, the other prototype (intradermal pen) was inferior. |
| Efficacy of the jet injector in local anaesthesia for small wound sutures: a randomised clinical trial compared with the needle infiltration technique | Saghi et al. | 2015 | Emerg Med J | Not Mentioned | Human (n=53) | NFJI Efficacy and Safety | RCT, Blinded, significantly less pain with NFJI compared to traditional needle-syringe but was longer to achieve anesthesia. |
| Needle-free jet injection for administration of influenza vaccine: a randomised non-inferiority trial | McAllister et al. | 2014 | Lancet | Spring (Stratis) | Human (n=1,250) | NFJI Efficacy | RCT - intra musclar flu vaccine, non-inferior to needle injection. Higher frequency of local adverse effects |
| A pilot study to examine the tolerability and device preference in type 1 diabetes of insulin aspart administered by InsuJet compared with subcutaneous injection | Reutens et al. | 2014 | Diabetes Technol Ther | Spring (Insujet) | Human (n=10) | NFJI Efficacy and Safety | Observational study - non-inferior insulin administration compared to traditional needle injection. Participant pain and tolerance was similar |
| Needle-free jet injection-induced small-droplet aerosol formation during intralesional bleomycin therapy | Bik et al. | 2021 | Lasers Surg Med | Pneumatic (EnerJet) & Spring (Dermojet) | Ex-Vivo Human Skin | NFJI Safety | NFJI produce small-particle aerosolized particles |
| A miniature shock wave driven micro-jet injector for needle-free vaccine/drug delivery | Batula et al. | 2016 | Biotechnol Bioeng | Pneumatic | Ex-Vivo Human Skin | NFJI Mechanics | Theoretical variable volumes and physics match actual volumes of injection, validating device |
| Needle-Free Jet Injection of Poly-(Lactic Acid) for Atrophic Acne Scars: Literature Review and Report of Clinical Cases | Rho et al. | 2024 | J Clin Md | Lorenz Force Actuator (CureJet) | Ex-Vivo Human Skin, Human (n=3) | NFJI Mechanics | Poly-Lactic injectable fillers injected into skin model to demonstrate consistency of injections |
| Delivery of immunoreactive antigen using a controllable needle-free jet injector | Hogan et al. | 2017 | J Control Release | Lorenz Force Actuator | Small Animal (Mouse Model) | NFJI Efficacy | Hepatitis B surface antigen injected intradermally by NFJI, 27g needle resulted in similar titers |
| Induction of potent antitumor immunity by intradermal DNA injection using a novel needle-free pyro-drive jet injector | Inoue et al. | 2023 | Cancer Sci | Pyro (Actranza) | Small Animal (Mouse Model) | NFJI Efficacy | intradermal efficacy of DNA vaccine was significant for NFJI, but non-existent for needle injection |
| Needle-Free Jet Injector-Assisted Triamcinolone Treatment of Keloids and Hypertrophic Scars is Effective and Well Tolerated in Children | Bekkers et al. | 2024 | Clin Drug Inestig | Pneumatic | Human (n=11) | NFJI Efficacy and Safety | Improved keloid scar appearance on POSAS, and lower median visual pain analog score compared to needle injections. |
| Subcutaneous nicotine delivery via needle-free jet injection: A porcine model | Ruddy et al. | 2019 | J Control Release | Lorenz Force Actuator | Large Animal (Porcine) | NFJI Efficacy | Systemic nicotine levels were similar with NFJI and needle injection |
| Needle-Free Injection of Metformin Ameliorates Skin Photoaging Through Inhibition of Ferroptosis and Oxidative Stress | Zhang et al. | 2024 | Discov Med | Not Specified | Small Animal (Rat Model) | NFJI Efficacy | Metformin injected intradermally reduced cellular senescence and decrease expression of reactive oxygen proteins |
| Estimation of High-Speed Liquid-Jet Velocity Using a Pyro Jet Injector | Takagaki et al. | 2019 | Sci Rep | Pyro | Ex-Vivo Human Skin | NFJI Mechanics | Theoretical variable volumes and physics match actual volumes of injection, validating device |
| Needle-free electronically-controlled jet injector treatment with bleomycin and lidocaine is effective and well-tolerated in patients with recalcitrant keloids | Bekkers et al. | 2024 | Lasers Surg Med | Pneumatic (EnerJet) | Human (n=15) | NFJI Efficacy and Safety | Improved keloid scar appearance on POSAS, high patient satisfaction scores and tolerable pain |
| Development of Pyro-Drive Jet Injector With Controllable Jet Pressure | Miyazaki et al. | 2019 | J Pharm Sci | Pyro (Acranza) | Large Animal (Porcine) | NFJI Mechanics | Porcine skin ex-vivo testing to confirm mathematic modeling of tissue penetration for each exit pressure and velocity |
| A liquid breakdown driven non-invasive microjet injection system | Ham et al. | 2019 | Med Eng Phys | Laser / Di-Electric | Ex-Vivo Porcine, Small Animal (Mouse) | NFJI Mechanics and Efficacy | Laser showed saturation of velocity with increasing power compared to di-electric. Insulin injected achieved higher insulin blood levels by the di-electric compared to laser NFJI. |
| Needle-free jet injection of intact phospholipid vesicles across the skin: a feasibility study | Schlich et al. | 2016 | Biomed Microdevices | Spring (Comfort-In) | Ex-Vivo Human Skin | NFJI Efficacy | Demonstrate the ability to deliver intact lipid vesicle formulation of medications |
| Non-Inferiority Field Study Comparing the Administrations by Conventional Needle-Syringe and Needle-Free Injectors of a Trivalent Vaccine Containing Porcine Circovirus Types 2a/2b and Mycoplasma hyopneumoniae | Cho et al. | 2022 | Vaccines | Pneumatic (Pulse FX) & Spring (EPIG) | Large Animal (Porcine) | NFJI Efficacy | Non-inferiority trial - NFJI delivered an adequate dose to achieve immunity for porcine vaccines |
| Dynamic behavior of a spring-powered micronozzle needle-free injector | Schoubben et al. | 2015 | Int J Pharm | Spring | Porcine skin | NFJI Mechanics | Ex-vivo investigation found direct dependence of force, velocity and duration with volume of injection |
| A Mathematical Model and Experimental Verification of Optimal Nozzle Diameter in Needle-Free Injection | Zeng et al. | 2018 | J Pharm Sci | Spring (Injex) | Small Animal (Mouse Model) | NFJI Mechanics and Efficacy | Insulin mouse model confirms theoretical calculations of varying nozzle diameters; found optimal intradermal dispersion with 0.30 mm nozzle diameter |
| Investigating skin penetration depth and shape following needle-free injection at different pressures: A cadaveric study | Seok et al. | 2016 | Lasers Surg Med | Pneumatic (Innojector) | Ex-Vivo Human Skin | NFJI Mechanics | Cadaver study showing how varying pressure ultimately affects shape of skin penetration and depth. High pressure injections create a smaller hole with deeper deposits. |
| Comparison of traditional anesthesia method and jet injector anesthesia method (MadaJet XL®) for Nexplanon® insertion and removal | Wilson et al. | 2020 | Contracept Reprod Med | Spring | Human (n=39) | NFJI Efficacy | Patient pain score lower with NFJI without compromise of anesthesia efficacy for Nexplanon(R) insertion/removal procedures |
| Needle-Free Injection Assisted Drug Delivery-Histological Characterization of Cutaneous Deposition | Erlendsson et al. | 2020 | Lasers Surg Med | Pneumatic (AirGent) | Porcine skin | NFJI Mechanics | Higher pressure setting had deeper deposition and point of maximal dispersion (reticular dermis) |
| Needle-free injectors for mass administration of fractional dose inactivated poliovirus vaccine in Karachi, Pakistan: A survey of caregiver and vaccinator acceptability | Daly et al. | 2020 | Vaccine | Spring (Tropis) | Human (n=5,508) | NFJI Patient Preference | Vaccine administrators and patient caregivers showed significantly higher preference of NFJI compared to needle-based vaccination techniques |
| HIV-1 vaccination by needle-free oral injection induces strong mucosal immunity and protects against SHIV challenge | Jones et al. | 2019 | Nat Commun | Pneumatic (Syrijet) | Large Animal (Monkey) | NFJI Efficacy | HIV1 vaccination effective through a NFJI trans-mucosal route of administration compared to standard topical mucosal absorption and subcutaneous injection |
| Human factors study of ZENEO® (needle-free autoinjector) and comparison of different user instruction formats | Allaet et al. | 2018 | Panmierva | Pneumatic (Zeneo) | Human (n=134) | NFJI Safety | Patients demonstrated proficient use of NFJI (no difference between reading material, video, dummy demonstration) during a simulation 6 weeks later. |
| Determining Losses in Jet Injection Subcutaneous Insulin Delivery: A Model-Based Approach | McHugh et al. | 2023 | J Diabetes Sci Technol | Not Specified | Human (n=7) | NFJI Efficacy | Approx. 22% of 2 unit insulin loss was observed with NFJI |
| Insulin delivery with a needle-free insulin injector versus a conventional insulin pen in Chinese patients with type 2 diabetes mellitus: A 16-week, multicenter, randomized clinical trial (the FREE study) | Ji et al. | 2020 | E Clinical Medicine | Spring (Beijing QS) | Human (n=412) | NFJI Efficacy and Safety | Prospective, multicenter, randomized trial. Superior HbA1c reduction and higher patient satisfaction/VAS scores with NFJI compared to traditional needle injection. |
| Carrier-free mRNA vaccine induces robust immunity against SARS-CoV-2 in mice and non-human primates without systemic reactogenicity | Abbasi et al. | 2024 | Mol Ther | Pyro | Small Animal (Mouse Model) | NFJI Efficacy | NFJI enhanced carrier-free mRNA vaccine efficiency due to increased immunogenicity with NFJI |
| High-Pressure Delivery of Oncolytic Viruses via Needle-Free Injection Preserves Therapeutic Activity | Said et al. | 2023 | Cancers | Spring (Inolife) | Small Animal (Mouse Model) | NFJI Efficacy | NFJI showed similar degree of tumor regression compared to needle injection delivery of viral oncolytics |
| Does the use of Dermojet affect the concentration of platelet-rich plasma? An in vitro experimental investigation | Gokkaya et al. | 2021 | Dermatol Therap | Spring (DermoJet) | Ex-Vivo Human Skin | NFJI Efficacy | Approx. 8% of platelets lost with PRP injection by the NFJI. |
| Toward jet injection by continuous-wave laser cavitation | Berrospe-Rodriguez et al. | 2017 | J Biomed Opt | Laser assisted | Ex-Vivo Human Skin | NFJI Mechanics | Continuous-wave assisted is a viable alternative power source for NFJI. Explored mechanics of jet injection velocity and depth of penetration. |
| Treatment of Palmar Hyperhidrosis With Needle Injection Versus Low-Pressure Needle-Free Jet Injection of OnabotulinumtoxinA: An Open-Label Prospective Study | Vadeboncoeur et al. | 2017 | Dermatol Surg | Pneumatic (Med-Jet) | Human (n=20) | NFJI Efficacy | NFJI was effective and not inferior to needle injection for the treatment of palmar hyperhidrosis with neurotoxin. NFJI injections were tolerable without prior anesthetic. |
| Home-Use Hyaluronic Acid Jet Injectors: Unreliable and Unsafe | Juch et al. | 2024 | Dermatol Surg | Pneumatic (EnerJet), Spring (Dermojet), Others | Ex-Vivo Human Skin | NFJI Efficacy | Home use NFJI showed markedly lower intradermal delivery of hyaluronic acid compared to medical grade NFJI injectors |
| Power-efficient controlled jet injection using a compound ampoule | McKeage et al. | 2018 | J Control Release | Lorenz Force Actuator | Porcine skin | NFJI Mechanics | A novel compound ampule with variable diameter within the chamber allows for two phases, ultimately decreasing amount of energy required to deliver 1mL of fluid |
| Relative Bioavailability Study of Midazolam Intramuscularly Administered with the Needle-Free Auto-Injector ZENEO® in Healthy Adults | Lacombe et al. | 2024 | Neurol Ther | Pneumatic (Zeneo) | Human (n=40) | NFJI Efficacy | IM midazolam via NFJI and needle-syringe showed equal bioavailability. Faster onset of medication effect seen with NFJI group |
| Clinical study of safety and immunogenicity of pentavalent DTP-HB-Hib vaccine administered by disposable-syringe jet injector in India | Bavdekar et al. | 2019 | Contemp Clin Trials Communic | Spring (Stratis) | Human (n=128) | NFJI Efficacy and Safety | Study stopped early due to moderate/severe injection reactions with NFJI |
| Colour marking of small fish with a marking stand for DermojetⓇ | Pitsh et al. | 2021 | MethodsX | Spring (DermoJet) | Small Animal (Fish Model) | NFJI Efficacy | Demonstrate the ability to quickly and reproducibly tattoo fish fins with blue dye using a NFJI |
| The effect of jet speed on large volume jet injection | McKeage et al. | 2018 | J Control Release | Lorenz Force Actuator | Porcine skin | NFJI Mechanics | Two phase jet injections are able to deliver similar amounts of fluid into the dermis with significantly less energy |
| Feasibility of jet injector use during inactivated poliovirus vaccine house-to-house vaccination campaigns | Farag et al. | 2018 | Vaccine | Spring (PharmaJet) | Human (n=993) | NFJI Safety | 91% of healthcare workers preferred / satisfied with needle-free safety element during high volume vaccination campaign |
| Facial Skin Rejuvenation Using Poly-dl-Lactic Acid Injected With a Laser-Generated Needle-Free Microjet Injector | Oh et al. | 2024 | Dermatol Surg | Laser assisted | Human (n=27) | NFJI Efficacy and Safety | Patients satisfied with NFJI delivery of PDLA. Skin improvements observed confirming efficacy of PDLA treatment. |
| Phase I Study to Assess the Safety and Immunogenicity of an Intradermal COVID-19 DNA Vaccine Administered Using a Pyro-Drive Jet Injector in Healthy Adults | Nakagami et al. | 2022 | Vaccines | Pyro | Human (n=20) | NFJI Efficacy and Safety | No safety issues identified. Mild/modest antibody response for both high dose and low dose group |
| Gene transfer by pyro-drive jet injector is a novel therapeutic approach for muscle diseases | Nakae et al. | 2021 | Gene | Pyro | Large Animal (Porcine) | NFJI Efficacy | Protein expression increased with NFJI injection of plasmid (gene therapy) compared to traditional needle-syringe technique. |
| Intravenous versus Subcutaneous Midazolam Using Jet-injector in Pediatric Sedation; a Randomized Clinical Trial | Hajimaghsoudi et al. | 2018 | Emerg (Tehran) | Spring (Injex30) | Human (n=60) | NFJI Efficacy | Intravenous and subcutaneous midazolam in children achieve equivalent amount of procedural sedation in children. Higher parent satisfaction with the subcutaneous sedation. |
| Degradation study on molecules released from laser-based jet injector | Krizek et al. | 2021 | Int J Pharm | Laser assisted (MiraJet) | Ex-Vivo Human Skin | NFJI Efficacy | DNA and Medications (ie. lidocaine) did not display any degradation or structural changes after delivery by NFJI |
| Comparison of Normal Saline Injection with Pneumatic Injector to Subcision for the Treatment of Atrophic Acne Scars | Pravangsuk et al. | 2021 | J Clin Aesthet Dermatol | Pneumatic (Innojector) | Human (n=18) | NFJI Efficacy | Prospective, randomized, blinded - normal saline and needle subsicions both improved visual severity of acne scars from baseline (no difference between treatments) |
| A compound ampoule for large-volume controllable jet injection | Ruddy et. al | 2015 | Annu Int Conf IEEE Eng Med Biol Sci | Lorenz Force Actuator | Large Animal (Porcine) | NFJI Mechanics | Double piston ampule allows for precise delivery of large volumes at greater depths |
| Potent Intradermal Gene Expression of Naked Plasmid DNA in Pig Skin Following Pyro-drive Jet Injection | Miyazaki et al. | 2021 | J Pharm Sci | Pyro (Actranza) | Porcine Skin | NFJI Efficacy | Intradermal injection of naked DNA with NFJI resulted in stable and more efficient gene expression compared to traditional needle injection |
| Immunogenic Comparison of Nucleic Acid-Based Vaccines Administered by Pyro-Drive Jet Injector | Tai et al. | 2024 | Vaccines | Pyro | Small Animal (Mouse Model) | NFJI Efficacy | NFJI used to test naked mRNA and naked DNA vaccination efficacy |
| Immune response induced in rodents by anti-Covid19 plasmid DNA vaccine via pyro-drive jet injector inoculation | Nishikawa et al. | 2022 | Immunol Med | Pyro | Small Animal (Mouse Model) | NFJI Efficacy | Pyro NFJI successfully inoculated mice with DNA plasmid vaccine for Covid-19 |
| Reduction in Lesion Incidence in Pork Carcass Using Transdermal Needle-free Injection of Foot-and-Mouth Disease Vaccine | Ko et al. | 2018 | Korean J Food Sci Anim Resour | Pneumatic | Large Animal (Porcine) | NFJI Efficacy and Safety | Significantly lower injection site damage/lesions with NFJI compared to needle-syringe |
| High-velocity pneumatic injection of non-crosslinked hyaluronic acid for skin regeneration and scar remodeling: A retrospective analysis of 115 patients | MacGillis et al. | 2021 | J Cosmet Dermatol | Pneumtc (EnerJet) | Human (n=115) | NFJI Efficacy and Safety | Similar clinical skin/scar improvement when highly crosslinked or non-crosslinked hyaluronic acid delivered with NFJI. Minimal injection site adverse effects |
| Investigating the use of local nerve blocks and general anaesthesia in reducing pain during and after disbudding procedure in goat kids | Cuttance et al. | 2023 | J Appl Anim Welf Sci | Not Specified | Large Animal (Goat) | NFJI Safety | NFJI were one of the variables for administration of local anesthetic. Local vs general anesthesia for disbudding procedures was being tested. |
| Effect of jet injection on infectivity of measles, mumps, and rubella vaccine in a bench model | Coughlin et al. | 2015 | Vaccine | Spring (Stratis) | Porcine Skin | NFJI Efficacy | Delivery of RNA based (live attenuated MMR) vaccine by NFJI did not compromise the structure and infectivity |
| Alternative vaccine administration by powder injection: Needle-free dermal delivery of the glycoconjugate meningococcal group Y vaccine | Weissmueller et al. | 2017 | PLos One | Pneumatic (Helium) | Small Animal (Mouse Model) | NFJI Efficacy | Clinical demonstration of successful vaccination with lyophilized meningococcal vaccination without aluminum adjuvant. Novel method to lyophilize vaccines. |
| The biodistribution of triamcinolone acetonide injections in severe keloids: an exploratory three-dimensional fluorescent cryomicrotome study | Yin et al. | 2024 | Arch Dermatol Res | Pneumatic | Human (n=30) | NFJI Efficacy | Triamcinolone injected in keloids by revealed highly variable distribution with both NFJI and traditional needle-syringe technique |
| Intradermal injection of a fractional dose of an inactivated HFMD vaccine elicits similar protective immunity to intramuscular inoculation of a full dose of an Al(OH)3-adjuvanted vaccine | Li et al. | 2017 | Vaccine | Pneumatic (Med-Jet) | Large Animal (Monkey) | NFJI Efficacy | Inactivated virus vaccine administered intradermally (one fourth the dose) by NFJI achieved the same degree of immunity as full dose administered intramuscularly with needle and syringe |
| Modified DNA vaccine confers improved humoral immune response and effective virus protection against SARS-CoV-2 delta variant | Hayashi et al. | 2022 | Sci Rep | Pyro | Small Animal (Mouse Model) | NFJI Efficacy | Found that levels of neutralizing antibodies induced by the intradermal PJI injection were higher than intramuscular injection by needle-syringe |
| Protective immunity induced by concurrent intradermal injection of porcine circovirus type 2 and Mycoplasma hyopneumoniae inactivated vaccines in pigs | Lee et al. | 2021 | Vaccine | Spring (IDAL) | Large Animal (Porcine) | NFJI Efficacy | Protective immune responses against PCV2 and Mhp could be efficiently induced in pigs using a relatively small volume of intradermal vaccines |
| Stable Immune Response Induced by Intradermal DNA Vaccination by a Novel Needleless Pyro-Drive Jet Injector | Chang et al. | 2019 | AAPS PharmSciTech | Pyro (Actranza) | Rat and Mouse Model | NFJI Efficacy | NFJI devices are an effective, novel method for delivery of plasmid DNA into epidermal and dermal cells suggesting its promise as a tool for DNA vaccination |
| Needle-free small-volume liquid injection system powered by a rotary actuator | Zhang et al. | 2017 | Annu Int Conf IEEE Eng Med Biol Sci | Screw Rotary | Ex-Vivo Human Skin | NFJI Mechanics | Introduce a compact, lightweight jet injector that uses a small commercial rotary motor and differential screw to create a jet of fluid with sufficient force to deliver drug into tissue |
| An experience of mass administration of fractional dose inactivated polio vaccine through intradermal needle-free injectors in Karachi, Sindh, Pakistan | Bullo et al. | 2021 | BMC Public Health | Spring (Tropis) | Human (n=33,818) | NFJI Efficacy | Higher vaccination rates with fractional inactive polio vaccine via NFJI compared to full dose needle-syringe |
| Delivery of Experimental mRNA Vaccine Encoding the RBD of SARS-CoV-2 by Jet Injection | Kisakov et al. | 2024 | Bull Exp Biol Med | Not Specified | Small Animal (Mouse Model) | NFJI Efficacy | NFJI of mRNA vaccine can be a efficaciously delivered and be an alternative to lipid nanoparticle delivery compared to traditional intra-muscular needle-syringe delivery |
| A clade C HIV-1 vaccine protects against heterologous SHIV infection by modulating IgG glycosylation and T helper response in macaques | Sahoo et al. | 2022 | Sci Immunol | Spring (Tropis) | Large Animal (Monkey) | NFJI Efficacy | Parenteral injection (NFJI) and oral were oth able to achieve a good antibody response for an HIV-1 vaccine |
| Intradermal administration of DNA vaccine targeting Omicron SARS-CoV-2 via pyro-drive jet injector provides the prolonged neutralizing antibody production via germinal center reaction | Hayashi et al. | 2023 | Sci Rep | Pyro | Small Animal (Rat Model) | NFJI Efficacy | Higher titers achieved with NFJI and DNA based covid vaccine for the omicron variant |
| Feasibility of conducting intradermal vaccination campaign with inactivated poliovirus vaccine using Tropis intradermal needle free injection system, Karachi, Pakistan | Yousafzai et al. | 2017 | Heliyon | Spring (Tropis) | Human (n=582) | NFJI Efficacy and Safety | No adverse events, no vaccine loss. Avg. 38 seconds per patient for injection administration (total, start to finish). Short learning curve exists. |
| Evaluation of Efficacy of Intraligamentary Injection Technique for Extraction of Mandibular Teeth-A Prospective Study | Pradhan et al. | 2017 | J Clin Diagn Res | Spring (Ligaject) | Human (n=194) | NFJI Efficacy | Intraligamentary dental injection with lidocaine and epinephrine delivered by NFJI demonstrated a decrease in injection pain and minimal procedural pain (molar extraction) |
| INJEX50 could improve the success rate of local anesthesia for arterial cannulation in the pediatric intensive care unit: A randomized, double-blind, single-center study | Sakai et al. | 2024 | Paediatr Anaesth | Spring (Injex50) | Human (n=70) | NFJI Efficacy | Randomized, double blind study demonstrating that NFJI showed significant improvement in anesthetic success in pediatric cases that require emergency arterial cannulalization |
| Factors Driving Patient Preferences for Growth Hormone Deficiency (GHD) Injection Regimen and Injection Device Features: A Discrete Choice Experiment | McNamara et al. | 2020 | Patient Prefer Adherence | Not Specified | Human (n=224) | Patient Preferences | Home growth hormone injection patients would prefer more infrequent injection schedule, |
| Long Term Follow-Up Study of a Randomized, Open-Label, Uncontrolled, Phase I/II Study to Assess the Safety and Immunogenicity of Intramuscular and Intradermal Doses of COVID-19 DNA Vaccine (AG0302-COVID19 | Nakagami et al. | 2023 | Vaccines | Pyro | Human (n=448) | NFJI Efficacy | Intradermal (with NFJI) and intramuscular Covid vaccine study of varying dosages show immune efficacy. |
| Intradermal Inoculation of Inactivated Foot-and-Mouth Disease Vaccine Induced Effective Immune Responses Comparable to Conventional Intramuscular Injection in Pigs | Lee et al. | 2024 | Vaccines | Laser assisted (MiraJet) | Large Animal (Porcine) | NFJI Efficacy | Intradermal (by NFJI) and intramuscular vaccine injections both demonstrated adequate immunity for hand-foot-mouth disease but fewer complications with intradermal injection. |
| Penetration and delivery characteristics of repetitive microjet injection into the skin | Romgens et al. | 2016 | J Control Release | Piezoelectric | Ex-Vivo Human and Porcine Skin | NFJI Mechanics | Microjets with a velocity exceeding 90m/s penetrated an epidermal skin sample with a delivery efficiency of approximately 96% and full-thickness skin with 12% efficiency. |
| Effective improvement methods for striae distensae: A novel approach utilizing laser-induced micro-jet injectors with poly-d,l-lactic acid | Seo et al. | 2024 | J Cosmet Dermatol | Laser assisted | Human (n=4) | NFJI Efficacy and Safety | Poly-d,l-lactic acid injections demonstrated effectiveness in treating skin stria when delivered via microjet injectors. Patients expressed high satisfaction with NFJI. |

NFJI (Needle Free Jet Injector), Comfort-In (Eternity Healthcare Inc, Vancouver, BC, Canada), MadaJet XL (Mada Medical Products Inc, Carlstadt, NJ, USA), Bejing QS (Beijing Med Tech Inc, Beijing, China), MedJet (MIT Canada, Montreal, Canada), Statis (PharmaJet, Golden, Colorado, USA), Tropis (PharmaJet, Golden, Colorado, USA), Bioject (Inovio, Plymouth Meeting, Pennsylvania, USA), AirGent (PerfAction, Tel Aviv, Israel), Prime (Portal Instruments, Cambridge, Massachusets, USA), Biojector (Bioject Medical Technologies, Bedminster, NJ, USA), Insujet (European Pharma Group, Schophol-Rijk, Netherlands), Zeneo (Crossject, Dijon, France)

Intradermal Pen (Bioject Medical Technologies, Bedminster, NJ, USA), Dermojet (Akra Dermojet, Pau, France), Enerjet (Sinclair, London, United Kingdom), Actranza (Daicel Corp, Tokyo, Japan), Pulse FX (Pulse Needle Free Systems Inc, Lenexa, Kansas, USA), EPIG (Henke Sass Wolf, Tuttlingen, Germany), Injex (Aijex Pharma, Toyoma, Japan), Innojector (Amore Pacific, Seoul, Korea), Syrijet (Keystone Industries, Cherry Hill, NJ, US), Inolife (Inolife R&D Inc., Toronto, Canada), Injex30 (Aijex Pharma, Toyoma, Japan), MiraJet (JSK Biomed, Seol, Korea), IDAL (Merk Animal Health, NJ, USA), Ligaject (Micro Mega, Besancon, France), Injex50 (Aijex Pharma, Toyoma, Japan), Pneumatic Jet Injector (Sansin, Nanchang City, Jiangxi, China)
